# Prognostic value and therapeutic potential of IAP family in head and neck squamous cell carcinoma

**DOI:** 10.18632/aging.205551

**Published:** 2024-02-15

**Authors:** Xiaoqian Yu, Weiwei Cao, Xuejie Yang, Canping Yu, Wenying Jiang, Hongbin Guo, Xiaoyun He, Cheng Mei, Chunlin Ou

**Affiliations:** 1 Department of Pathology, Xiangya Hospital, Central South University, Changsha 410008, Hunan, China; 2 Department of Blood Transfusion, Xiangya Hospital, Clinical Transfusion Research Center, Central South University, Changsha 410008, Hunan, China; 3 Department of Orthopedics, Xiangya Hospital, Central South University, Changsha 410008, Hunan, China; 4 Departments of Ultrasound Imaging, Xiangya Hospital, Central South University, Changsha 410008, Hunan, China; 5 National Clinical Research Center for Geriatric Disorders, Xiangya Hospital, Central South University, Changsha 410008, Hunan, China

**Keywords:** IAP family, head and neck squamous cell carcinoma, prognosis, immune cells, immune targets

## Abstract

Head and neck squamous cell carcinoma (HNSCC) ranks as the eighth most prevalent malignancy globally and has the eighth greatest fatality rate when compared to all other forms of cancer. The inhibitor of apoptosis protein (IAP) family comprises a collection of apoptosis-negative modulators characterized by at least one single baculovirus IAP repeat (BIR) domain in its N-terminal region. While the involvement of the IAP family is associated with the initiation and progression of numerous tumours, its specific role in HNSCC remains poorly understood. Thus, this study aimed to comprehensively examine changes in gene expression, immunomodulatory effects, prognosis, and functional enrichment of HNSCC utilising bioinformatics analysis. Elevated levels of distinct IAP family members were observed to varying degrees in HNSCC, with high BIRC2 expression indicating a worse prognosis. Additionally, Gene Ontology and the Kyoto Encyclopedia of Genes and Genomes (KEGG) were used to probe the enrichment of gene expression and biological processes related to the IAP family in HNSCC. The infiltration levels of immune cells were shown to be strongly associated with the IAP gene expression, as determined by subsequent analysis. Hence, BIRC2 could be an effective immunotherapy target for HNSCC. Collectively, novel knowledge of the biological roles and prognostic implications of IAP family members in HNSCC is presented in this study.

## INTRODUCTION

Head and neck squamous cell carcinoma (HNSCC) ranks eighth in both the prevalence and death rate of all malignancies globally [1]. HNSCC arises from the mucosal epithelial cells lining the oral cavity, pharyngeal, laryngeal, and sinonasal tracts. The histological progression to invasive HNSCC is sequential, beginning with hyperplastic epithelial cells, followed by dysplastic cells (mild, moderate, and severe), carcinoma *in situ*, and finally, invasive carcinoma [2]. Recently, multiple studies have reported an increase in the incidence of HNSCC [3]. In most cases of HNSCC, the disease has already progressed to a locally advanced stage, necessitating multimodal therapy. Despite this approach’s therapeutic purpose, a large proportion of patients will experience locoregional failure and/or distant metastases [4]. Therefore, novel biomarkers and immunotherapy targets must be identified to improve outcomes [5, 6].

The inhibitor of apoptosis protein (IAP) family comprises a group of apoptosis-negative regulators characterised by at least one copy of the baculovirus IAP repeat (BIR) domain in the N-terminal region [7]. These proteins were first discovered in viruses because of their effect on host cells and were named apoptosis-inhibitory proteins [8]. Eight members make up the IAP family: testis-specific IAP (Ts-IAP/BIRC8), livin/BIRC7, BIR repeat-containing ubiquitin-conjugating enzyme (BRUCE/BIRC6), X-linked IAP (XIAP/BIRC4), survivin/BIRC5, cIAP2/BIRC3, cellular IAPs (cIAP1/BIRC2), and neuronal IAP (NAIP/BIRC1). Research has revealed that the IAP family plays a role in cell cycle regulation, cell migration, and other biological processes [9–11]. Aberrant expression of its members is linked to the development of tumours [12–15] and can serve as tumour markers [16, 17].

However, there have been no comprehensive analyses of IAP family expression, prognosis, or immune properties in HNSCC. Herein, bioinformatics technologies and research databases were used to analyse the link between IAP family gene expression and clinical characteristics in HNSCC. Our findings offer novel perspectives on the biological functions and prognostic relevance of IAP family members in HNSCC.

## RESULTS

### Aberrant expression of the IAP family members in HNSCC

**Figure 1:**
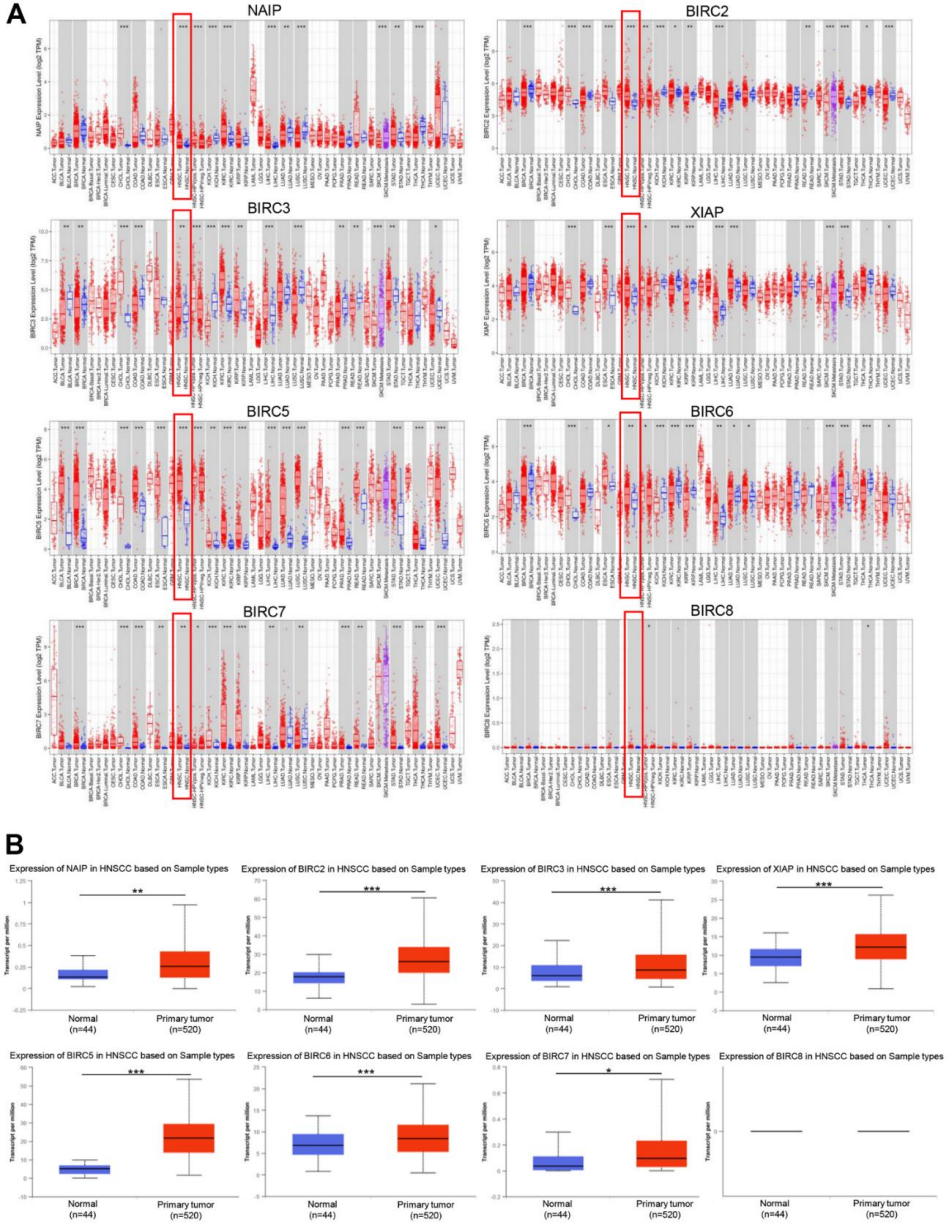
**The expression of IAP family members in HNSCC.** (**A**) The IAPs expression in pan-cancer. (**B**) IAPs expression in HNSCC. *P < 0.05, **P < 0.01, and ***P < 0.001 relative to that in controls.

Using the TIMER2.0 database, we examined IAP transcript levels to determine if there were any variations in their expression levels between normal and tumour samples. Notably, the mRNA expression levels of XIAP, *BIRC3, BIRC2, NAIP,* and *BIRC5-7* were markedly upregulated in HNSCC tissues. However, BIRC8 expression showed no statistically significant differences (Figure 1A). The University of Alabama at Birmingham Cancer Data Analysis (UALCAN) portal (https://ualcan.path.uab.edu) provides easy access to precalculated gene and protein expression based on tumour subgroups. This comprehensive web resource was employed to delve deeper into the analysis of the IAP family genes’ mRNA expression. In comparison to normal tissues, HNSCC tissues exhibited a significant upregulation of all IAPs except BIRC8 (*P* < 0.05) (Figure 1B).

**Figure 2:**
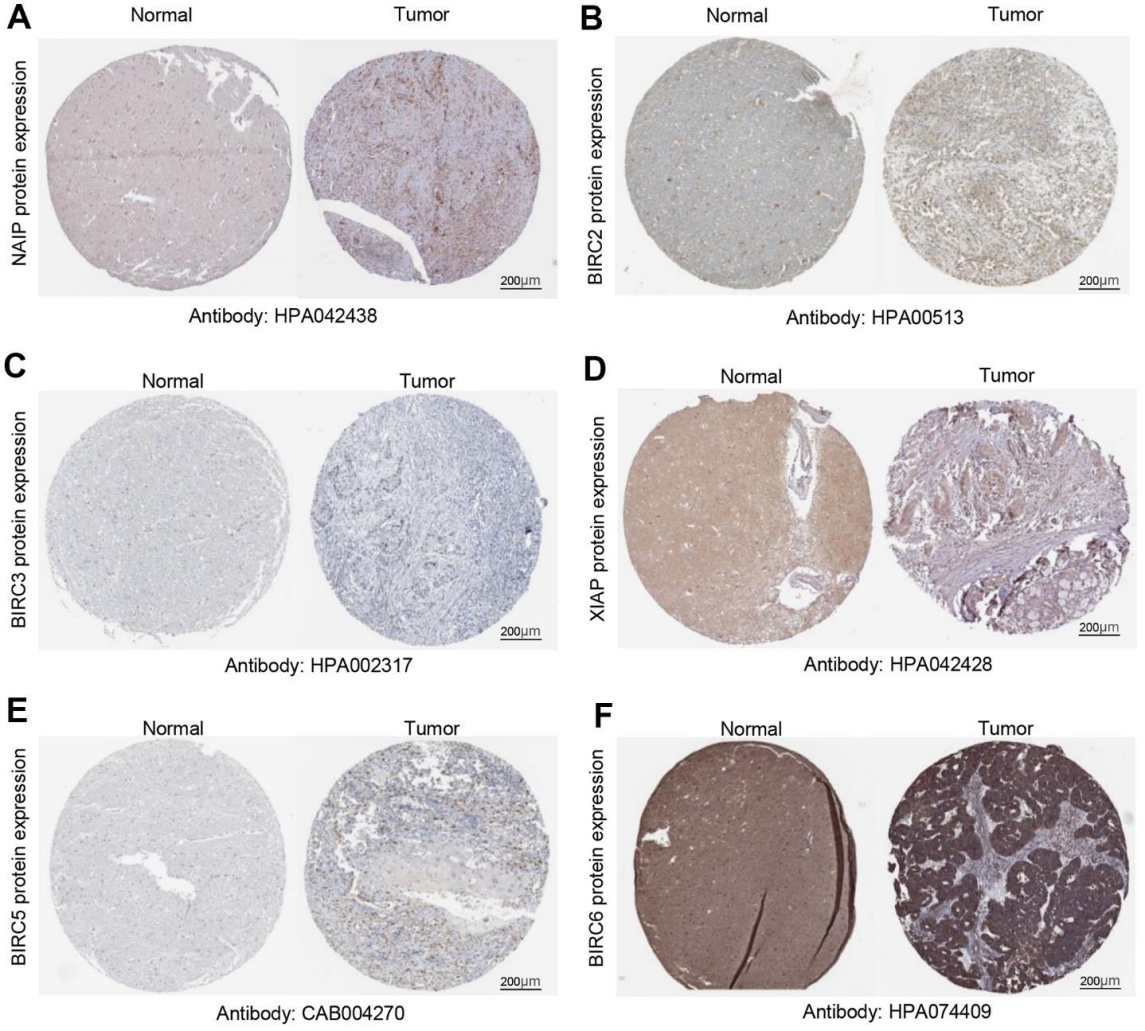
**Quantification of IAP family protein expression in HNSCC.** (**A**–**F**) Protein expression levels of BIRC6, BIRC3, BIRC2, XIAP, BIRC5, and NAIP in HNSCC relative to those of non-cancerous tissues.

We verified these findings by delving deeper into the HPA database for immunohistochemistry results from the IAP family. Normal tissues had lower protein levels of NAIP, BIRC2/3/5/6, and XIAP compared to HNSCC (Figure 2A–2F). Our previous findings on the IAP family mRNA expression levels are corroborated by these results.

### Correlation of IAP family expression with clinicopathologic features of HNSCC

**Figure 3:**
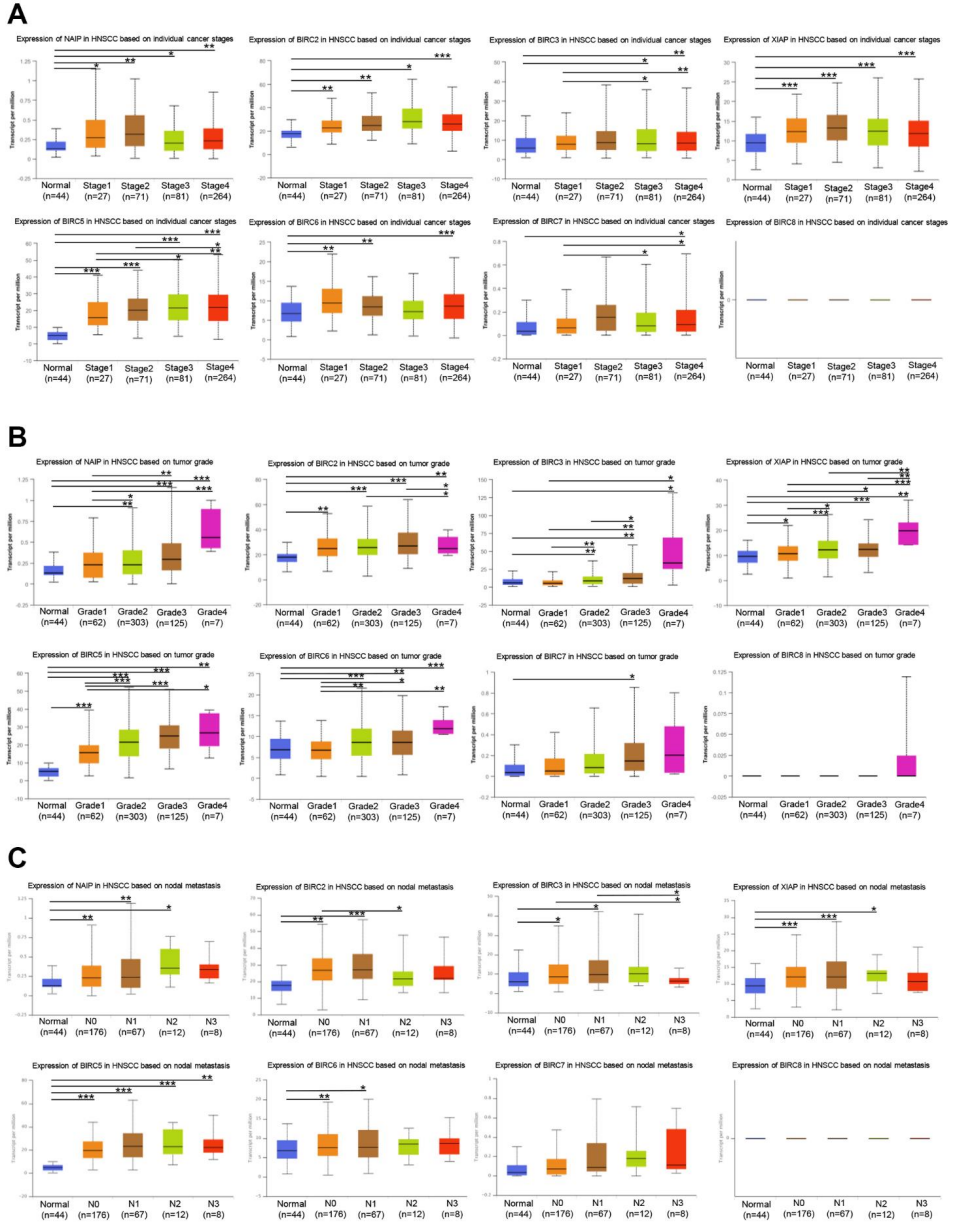
**Association of stage, grade, and lymph node metastasis of HNSCC with IAP family members.** (**A**) Association between stage of HNSCC patients and the mRNA expression levels of the IAP family. (**B**) Correlation between the grade of HNSCC patients and the mRNA levels of the IAP family. (**C**) The correlation between lymph node metastases and levels of IAP family mRNA expression in HNSCC patients. *P < 0.05, **P < 0.01, and ***P < 0.001 vs that between IAP family MRNA expression levels and control.

Next, we aimed to determine whether the IAP family members’ expression levels were linked to staging, grading, and lymph node metastasis of HNSCC. Seven IAP family genes (except BIRC8) exhibited elevated mRNA levels in the tumour stage 1–4 subgroups relative to normal tissue, according to the findings. NAIP, BIRC2/5, and XIAP expression were related to the individual cancer stages of HNSCC (Figure 3A). The BIRC2, XIAP, and BIRC5 mRNA expression levels were markedly linked to the individual HNSCC cancer grades as shown in Figure 3B. We found a variable degree of correlation between NAIP, BIRC2/3/5/6, and XIAP, and lymph node metastasis in HNSCC (Figure 3C). Based on these results, IAPs (particularly BIRC2/5 and XIAP) contribute to the onset and progression of HNSCC.

### Prognostic value of IAP gene family in patients with HNSCC

**Figure 4:**
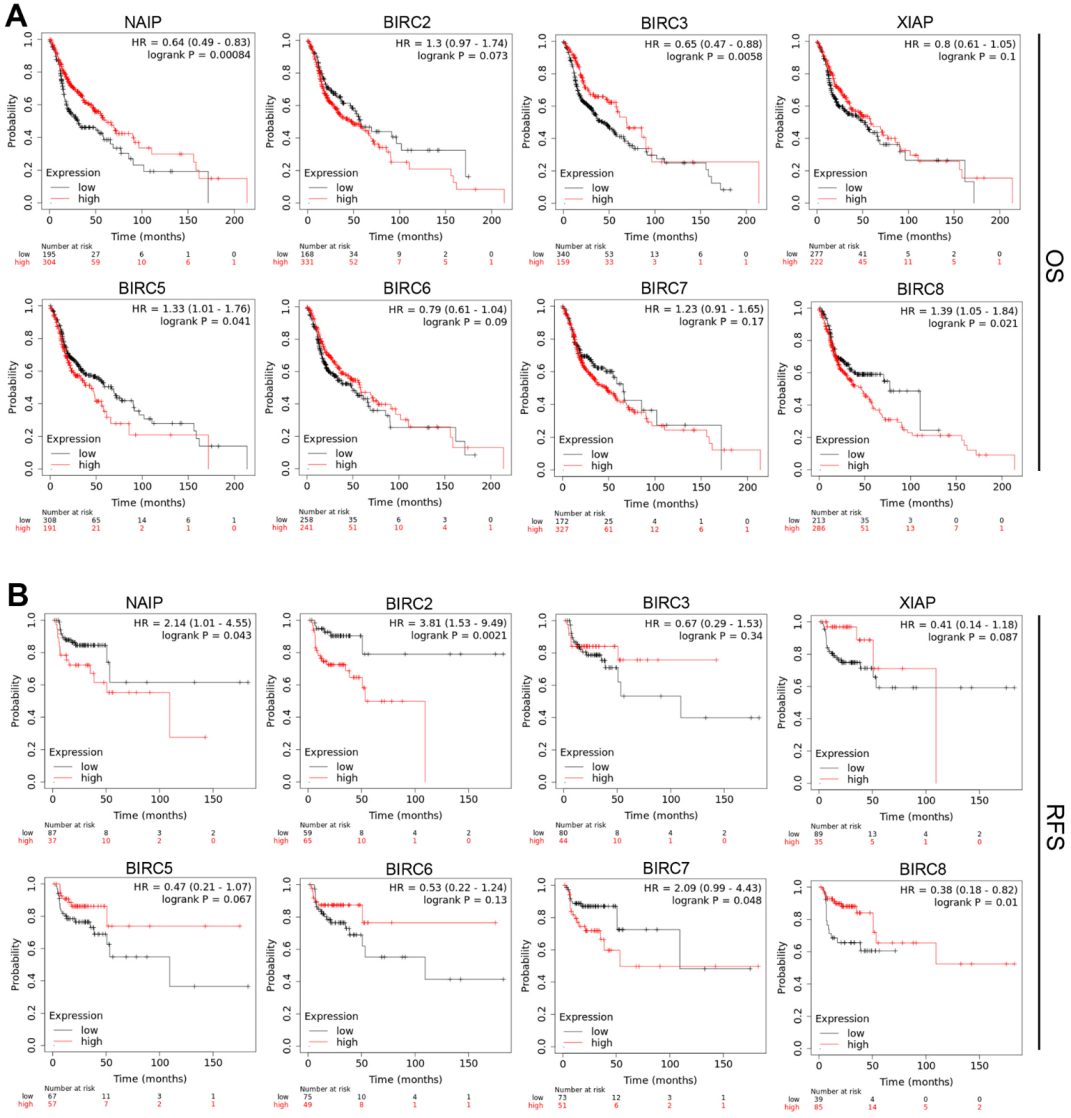
**Prognostic significance of IAP family members’ mRNA expression levels in HNSCC patients.** (**A**, **B**) The OS and RFS of the IAP family in HNSCC were analysed utilising a Kaplan–Meier plot.

IAP family members’ predictive significance was evaluated in patients with HNSCC depending on their mRNA expression. This analysis was performed via the Kaplan–Meier (KM) plotter database. To ascertain the clinical prognostic outcome, survival analysis was performed, involving both recurrence-free survival (RFS) and overall survival (OS). According to the analysis, elevated mRNA levels of BIRC5 [OS: hazard ratio (HR) = 1.33 (1.01–1.76), *P* = 0.041] and BIRC8 [OS: HR = 1.39 (1.05–1.84), *P* = 0.021] were linked to a dismal OS in patients with HNSCC (Figure 4A). BIRC2 upregulation was linked to unfavorable OS; Still, the variation did not reach statistical significance. Increased expression of NAIP [RFS: HR = 2.14 (1.01–4.55), *P* = 0.043], BIRC2 [RFS: HR = 3.81 (1.53–9.49), *P* = 0.0021], and BIRC7 [RFS: HR = 2.09 (0.99–4.43), *P* = 0.048] was related to unfavorable RFS in HNSCC patients (Figure 4B).

### Genetic alteration and functional analysis of the IAP family in HNSCC individuals

**Figure 5:**
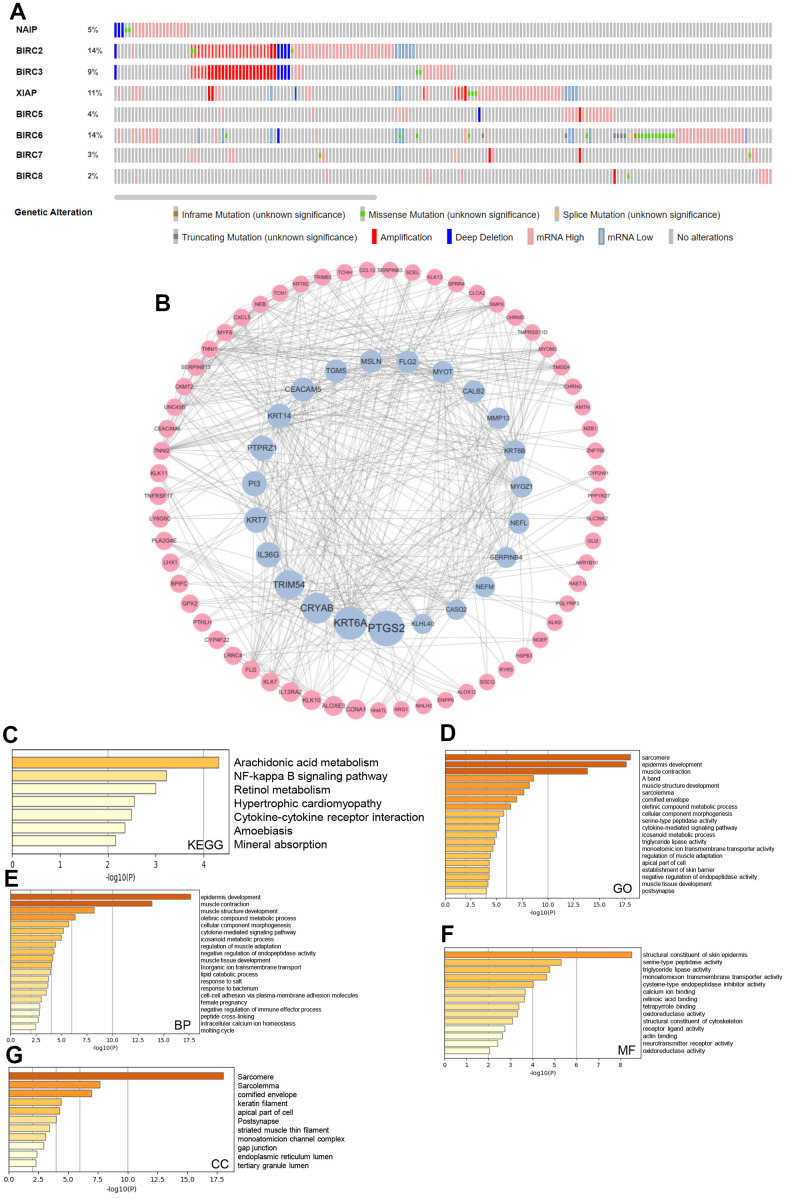
**IAP family genetic alterations in HNSCC and pathway enrichment analysis.** (**A**) IAP family alteration rates summarised by cBioPortal. (**B**) The cBioPortal database was utilised to find the 156 IAP-related co-expressed molecules that undergo the highest frequency of alteration in HNSCC. Members of the IAP family and the co-expressed genes linked with them were employed to design the PPI network. The Cytoscape database was applied in the establishment of the network. (**C**–**G**) Function enrichment analysis was performed to examine the biological roles of the IAP members and the genes they co-expressed.

Mutations are integral to cancer development, and some of these mutations can create specific vulnerabilities [18]. We investigated the genetic alterations in individual IAP family members using the cBioPortal dataset. Alterations were seen in all eight members of the IAP family among patients diagnosed with HNSCC, with respective alteration rates of 5, 14, 9, 11, 4, 14, 3, and 2 %. Additionally, mRNA alterations constituted the most prevalent forms of aberrations within the IAP family, with amplifications and deep deletions ranking second and third, respectively (Figure 5A). Next, cBioPortal was utilised to identify co-expressed genes with a threshold of |log2 fold-change| ≥ 0.5 and *P* < 0.05 (Supplementary Table 1), and Cytoscape v3.9.0 was utilised to produce a visualisation of the co-expression network of crucial genes that are related to the IAP family (Figure 5B). Metascape was utilised to evaluate the biological activities of the IAP members and their co-expressed genes by means of Kyoto Encyclopaedia of Genes and Genomes (KEGG) pathway analysis and Gene Ontology (GO) annotation. Using KEGG pathway analysis, the co-expressed genes were examined for their involvement in arachidonic acid metabolism, cytokine-cytokine receptor interactions, and the NF-kappa B signalling pathway (Figure 5C). The GO analysis illustrated the co-expression of genes primarily linked to the sarcomere, epidermal development, and cytokine-mediated signalling pathways (Figure 5D). Epidermal development, muscular contraction, and cytokine-mediated signalling pathways comprised the majority of the functions of these genes, according to bioprocess analysis. (Figure 5E). Additionally, the molecular function analysis revealed the involvement of these genes primarily in the structural components of the epidermis of the skin, serine peptidase activity, and triglyceride lipase activity (Figure 5F). The examination of cellular components demonstrated that these genes had a high degree of association with the cornified envelope and sarcomere (Figure 5G).

### Association of expression of IAP family members with immune infiltration in HNSCC

**Figure 6:**
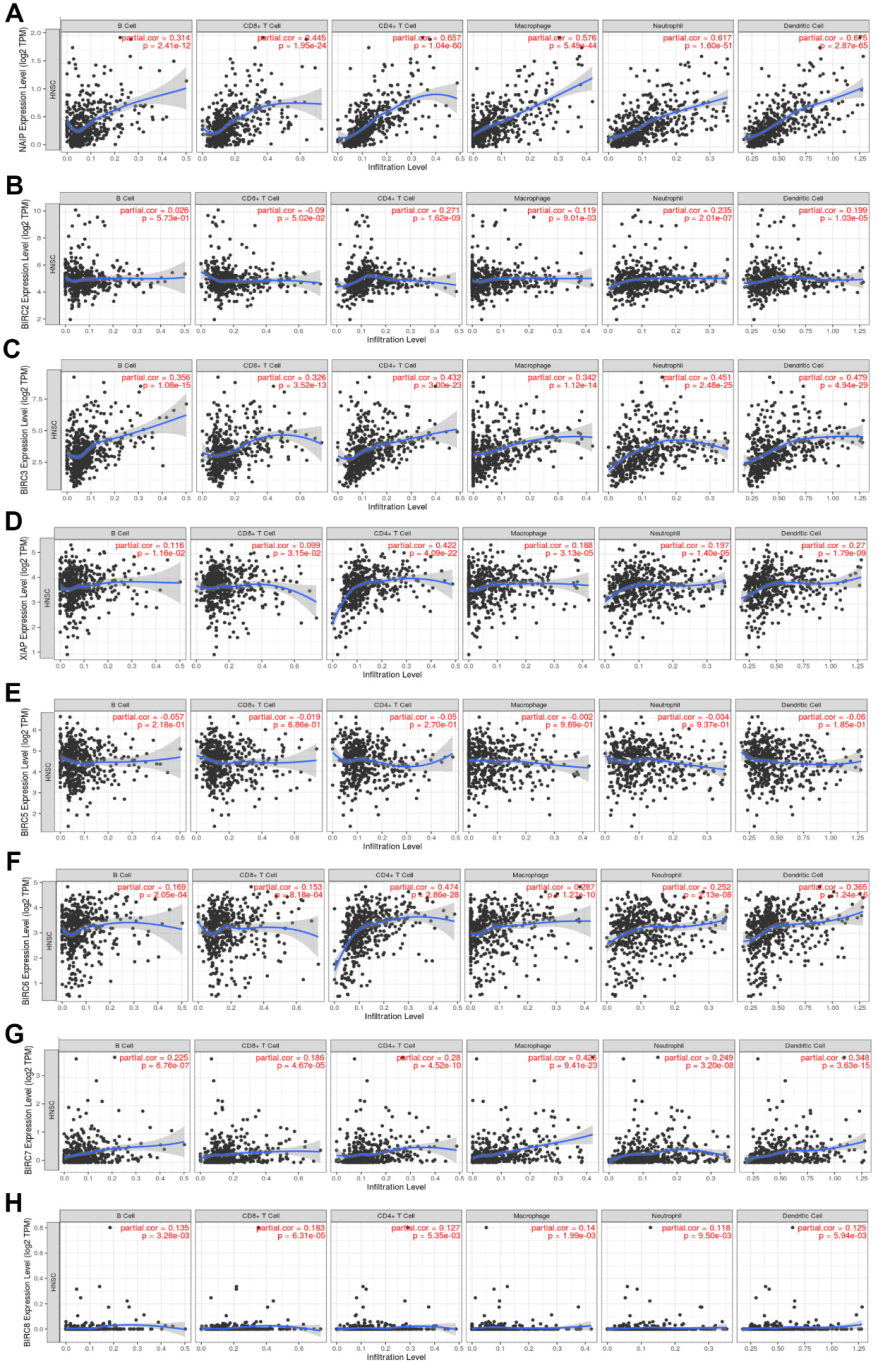
**Association between the level of IAP mRNA expressed and the infiltration of immune cells.** (**A**–**H**) Immune cell infiltration was assessed by examining the relationships between IAP family members and the TIMER database.

The Tumour Immune Estimation Resource (TIMER) was employed to determine the link between immune cell infiltration and the IAP gene family (Figure 6). The results illustrated that NAIP expression was substantially linked to B-cell (cor = 0.314, *P* < 0.05), CD8^+^ T-cell (cor = 0.445, *P* < 0.05), CD4^+^ T-cell (cor = 0.657, *P* < 0.05), macrophage (cor = 0.576, *P* < 0.05), neutrophil (cor = 0.717, *P* < 0.05), and dendritic cell (cor = 0.675, *P* < 0.05) infiltration. There was a strong link between BIRC2 mRNA expression and the infiltrating levels of CD4^+^ T-cells (cor = 0.271, *P* < 0.05), macrophages (cor = 0.119, *P* < 0.05), neutrophils (cor = 0.235, *P* < 0.05), and dendritic cells (DCs) (cor = 0.199, *P* < 0.05). Significant associations were observed between BIRC3 expression and B-cell (cor = 0.356, *P* < 0.05), CD8^+^ T-cell (cor = 0.326, *P* < 0.05), CD4^+^ T-cell (cor = 0.432, *P* < 0.05), macrophage (cor = 0.342, *P* < 0.05), neutrophil (cor = 0.451, *P* < 0.05), and DCs (cor = 0.479, *P* < 0.05) infiltrates. Significant associations were observed between the levels of B-cells (cor = 0.116, *P* < 0.05), CD8^+^ T-cells (cor = 0.099, *P* < 0.05), CD4^+^ T-cells (cor = 0.422, *P* < 0.05), macrophages (cor = 0.188, *P* < 0.05), neutrophils (cor = 0.197, *P* < 0.05), and DCs (cor = 0.270, *P* < 0.05). There was a strong link between BIRC6 mRNA expression and the infiltrating levels of B-cells (cor = 0.169, *P* < 0.05), CD8^+^ T-cells (cor = 0.153, *P* < 0.05), CD4^+^ T-cells (cor = 0.474, *P* < 0.05), macrophages (cor = 0.287, *P* < 0.05), neutrophils (cor = 0.252, *P* < 0.05), and DCs (cor = 0.365, *P* < 0.05). Considerable associations were observed between BIRC7 expression and B-cell (cor = 0.225, *P* < 0.05), CD8^+^ T-cell (cor = 0.186, *P* < 0.05), CD4^+^ T-cell (cor = 0.280, *P* < 0.05), macrophage (cor = 0.426, *P* < 0.05), neutrophil (cor = 0.249, *P* < 0.05), and DCs (cor = 0.348, *P* < 0.05) infiltrates. The level of BIRC8 mRNA was substantially linked to B-cell (cor = 0.135, *P* < 0.05), CD8^+^ T-cell (cor = 0.183, *P* < 0.05), CD4^+^ T-cell (cor = 0.127, *P* < 0.05), macrophage (cor = 0.140, *P* < 0.05), neutrophil (cor = 0.118, *P* < 0.05), and DC (cor = 0.125, *P* < 0.05) infiltrates. However, immune cell infiltration did not exhibit a link to BIRC5 expression. In the HNSCC tumour microenvironment (TME), our data indicate that the IAP family members, including BIRC2, may affect immune response.

The marker types present in HNSCC were examined by the TIMER database, including DCs, CD8+ T lymphocytes, neutrophils, T helper 1 (Th1) cells, and tumour-associated macrophages (TAM). This study aimed to examine immune cell-IAP family expression relationships (Table 1). A strong association exists between the levels of immune cells and members of the IAP family, except for BIRC5. Moreover, NAIP was shown to be strongly linked to CD8+ T lymphocytes, B, and TAMs, M2 macrophages, neutrophils, DCs, natural killer cells (NK), Th1, Th2, Tfh, Th17, regulatory T cells (Tregs), exhausted T cells, and monocytes. Additionally, BIRC2 exhibited a good correlation with TAMs, Tregs, M2 macrophages, DCs, Th1, Th2, and monocytes. BIRC3 demonstrated a robust correlation with CD8+ T lymphocytes, B cells, TAM, M1/M2 macrophages, monocytes, DCs, NK cells, exhausted T cells, Th1 cells, regulatory T cells, Th2 cells, T follicular helper cells, Th17 cells, and neutrophils. XIAP exhibited a significant association with B cells, T cells, TAM, M1/M2 macrophages, neutrophils, DCs, NK cells, Th1 cells, Th2 cells, T follicular helper cells, Th17 cells, regulatory T cells, exhausted T cells, and monocytes. Moreover, BIRC6 demonstrated a positive relationship with TAMs, M1/M2 macrophages, neutrophils, DCs, NK cells, Th1 cells, Th2 cells, monocytes, exhausted T cells, Th17 cells, regulatory T cells, and T follicular helper cells. There is a significant connection between BIRC7 and CD8+ T lymphocytes, T cells, TAMs, exhausted T cells, neutrophils, DCs, B cells, NK, Th1, Th2, Tfh, Th17, Tregs, M1/M2 macrophages, and monocytes. Additionally, BIRC8 displays a strong correlation with T cells, CD8+ T lymphocytes, B cells, TAMs, DC, NK, and Th1 cells. These observations indicate that the IAP family members may have contributed to the immune infiltration in HNSCC.

**Table 1:** The correlations between the expression of IAP family members and the markers of immune cells.

		**NAIP**		**BIRC2**		**BIRC3**		**XIAP**		**BIRC5**		**BIRC6**		**BIRC7**		**BIRC8**	
		**Cor**	***P***	**Cor**	***P***	**Cor**	***P***	**Cor**	***P***	**Cor**	***P***	**Cor**	***P***	**Cor**	***P***	**Cor**	***P***
CD8+ Tcell	CD8A	0.557	***	-0.029	0.527	0.386	***	0.141	**	-0.024	0.600	0.202	***	0.236	***	0.194	***
	CD8B	0.529	***	-0.072	0.11	0.385	***	0.066	0.147	0.012	0.789	0.129	**	0.265	***	0.206	***
	GZMA	0.411	***	0.018	0.696	0.303	***	0.023	0.615	0.054	0.234	0.052	0.252	0.251	***	0.148	**
B cell	CD19	0.451	***	-0.016	0.722	0.445	***	0.205	***	0.052	0.251	0.255	***	0.191	***	0.202	***
	CD79A	0.439	***	-0.011	0.807	0.419	***	0.226	***	0.024	0.593	0.318	***	0.107	*	0.180	***
	MS4A1	0.501	***	0.004	0.924	0.440	***	0.258	***	-0.033	0.463	0.308	***	0.150	**	0.154	**
T cell	CD3D	0.485	***	-0.041	0.36	0.416	***	0.049	0.282	0.050	0.267	0.081	0.074	0.286	***	0.198	***
	CD3E	0.611	***	0.013	0.772	0.466	***	0.201	***	-0.033	0.462	0.257	***	0.289	***	0.199	***
	CD2	0.603	***	0.001	0.988	0.435	***	0.171	***	-0.014	0.750	0.21	***	0.324	***	0.194	***
TAM	CCL2	0.524	***	0.179	***	0.301	***	0.292	***	-0.003	0.941	0.359	***	0.233	***	0.101	*
	CD68	0.391	***	0.048	0.291	0.189	***	0.315	***	-0.068	0.135	0.356	***	0.187	***	0.031	0.495
	IL10	0.546	***	0.252	***	0.335	***	0.309	***	-0.057	0.208	0.321	***	0.172	***	0.028	0.542
M1	IRF5	0.351	***	-0.039	0.389	0.280	***	0.239	***	-0.083	0.065	0.309	***	0.196	***	0.113	*
	PTGS2	0.005	0.912	0.278	***	0.144	**	0.236	***	0.036	0.421	0.217	***	-0.250	***	0.014	0.759
	NOS2	0.365	***	0.044	0.329	0.348	***	0.278	***	0.067	0.136	0.353	***	0.088	0.051	0.138	**
M2	MS4A4A	0.604	***	0.181	***	0.344	***	0.163	***	0.037	0.409	0.299	***	0.383	***	0.072	0.109
	CD163	0.645	***	0.220	***	0.369	***	0.244	***	0.026	0.568	0.403	***	0.279	***	0.042	0.351
	VSIG4	0.567	***	0.172	***	0.316	***	0.172	***	0.031	0.489	0.304	***	0.344	***	0.063	0.161
Neutrophils	ITGAM	0.594	***	0.149	**	0.459	***	0.310	***	-0.021	0.638	0.421	***	0.266	***	0.155	**
	CCR7	0.603	***	0.059	0.192	0.500	***	0.289	***	-0.046	0.311	0.340	***	0.218	***	0.161	***
	SIGLEC5	0.735	***	0.219	***	0.501	***	0.371	***	0.067	0.135	0.511	***	0.225	***	0.148	**
DC	HLA-DQB1	0.435	***	0.077	0.090	0.357	***	0.105	*	-0.004	0.927	0.158	***	0.324	***	0.139	**
	HLA-DPB1	0.578	***	0.045	0.318	0.414	***	0.124	**	-0.047	0.302	0.193	***	0.355	***	0.151	**
	HLA-DRA	0.610	***	0.125	**	0.455	***	0.181	***	-0.063	0.161	0.258	***	0.276	***	0.132	**
	HLA-DPA1	0.621	***	0.114	*	0.434	***	0.194	***	-0.077	0.088	0.286	***	0.289	***	0.135	**
	ITGAX	0.647	***	0.179	***	0.430	***	0.291	***	0.032	0.475	0.396	***	0.412	***	0.166	***
	CD1C	0.454	***	0.059	0.192	0.302	***	0.267	***	-0.220	***	0.272	***	0.258	***	0.102	*
	NRP1	0.523	***	0.345	***	0.193	***	0.354	***	-0.079	0.082	0.503	***	0.155	**	-0.035	0.437
NK cell	KIR2DL1	0.237	***	0.080	0.075	0.267	***	0.022	0.626	-0.025	0.586	0.104	*	0.059	0.192	0.067	0.140
	KIR2DL3	0.357	***	-0.013	0.780	0.269	***	0.067	0.137	0.002	0.959	0.116	*	0.134	**	0.089	*
	KIR2DL4	0.372	***	0.004	0.937	0.282	***	0.098	*	0.002	0.973	0.162	***	0.138	**	0.160	***
	KIR3DL1	0.390	***	-0.067	0.136	0.240	***	0.113	*	-0.016	0.723	0.190	***	0.084	0.063	0.163	***
	KIR3DL2	0.441	***	0.033	0.466	0.378	***	0.178	***	-0.048	0.287	0.233	***	0.166	***	0.168	***
	KIR3DL3	0.154	**	-0.054	0.229	0.158	***	0.066	0.143	0.050	0.270	0.052	0.252	0.135	**	0.211	***
	KIR2DS4	0.224	***	-0.080	0.075	0.158	***	0.043	0.340	-0.013	0.778	0.081	0.071	0.096	*	0.045	0.318
Th1	TBX21	0.567	***	-0.028	0.538	0.379	***	0.147	**	0.024	0.599	0.206	***	0.309	***	0.200	***
	STAT1	0.445	***	0.220	***	0.266	***	0.229	***	-0.068	0.134	0.280	***	0.099	*	0.028	0.538
	STAT4	0.549	***	0.246	***	0.486	***	0.216	***	0.024	0.588	0.262	***	0.329	***	0.116	*
	IFNG	0.411	***	-0.030	0.511	0.282	***	0.037	0.418	0.030	0.514	0.037	0.412	0.222	***	0.156	***
Th2	STAT6	0.419	***	0.217	***	0.290	***	0.524	***	0.017	0.705	0.561	***	0.011	0.813	0.074	0.103
	GATA3	0.348	***	0.095	*	0.173	***	0.147	**	-0.179	***	0.179	***	0.220	***	0.064	0.160
	STAT5A	0.567	***	0.157	***	0.564	***	0.235	***	0.074	0.100	0.319	***	0.240	***	0.137	**
Tfh	BCL6	0.378	***	0.124	**	0.221	***	0.480	***	-0.115	*	0.568	***	0.105	*	0.084	0.063
	IL21	0.456	***	0.055	0.225	0.330	***	0.258	***	-0.051	0.257	0.308	***	0.106	*	0.162	***
Th17	STAT3	0.501	***	0.235	***	0.425	***	0.534	***	-0.097	*	0.681	***	-0.005	0.915	0.072	0.112
	IL17A	0.299	***	-0.064	0.157	0.198	***	0.213	***	-0.019	0.667	0.180	***	0.009	0.837	0.016	0.729
Treg	FOXP3	0.714	***	0.154	**	0.470	***	0.396	***	-0.088	0.050	0.471	***	0.245	***	0.144	**
	STAT5B	0.570	***	0.273	***	0.336	***	0.496	***	-0.019	0.675	0.633	***	0.174	***	0.064	0.155
	CCR8	0.735	***	0.264	***	0.453	***	0.516	***	-0.107	*	0.639	***	0.156	**	0.120	**
T exhaustion-cell	PDCD1	0.556	***	-0.011	0.816	0.425	***	0.121	**	0.023	0.612	0.167	***	0.295	***	0.212	***
	CTLA4	0.560	***	0.121	**	0.477	***	0.136	**	0.074	0.103	0.156	***	0.329	***	0.152	**
	HAVCR2	0.663	***	0.160	***	0.428	***	0.217	***	0.035	0.444	0.308	***	0.411	***	0.156	**
	LAG3	0.480	***	0.023	0.606	0.354	***	0.018	0.685	0.116	*	0.070	0.122	0.280	***	0.182	***
Monocyte	CD86	0.606	***	0.252	***	0.454	***	0.223	***	0.018	0.695	0.330	***	0.345	***	0.086	0.057
	C3AR1	0.680	***	0.213	***	0.406	***	0.238	***	0.021	0.635	0.343	***	0.351	***	0.088	0.052
	CSF1R	0.705	***	0.207	***	0.425	***	0.261	***	-0.063	0.163	0.383	***	0.349	***	0.110	*

*P < 0.05.

**P < 0.01.

***P < 0.001.

**Figure 7:**
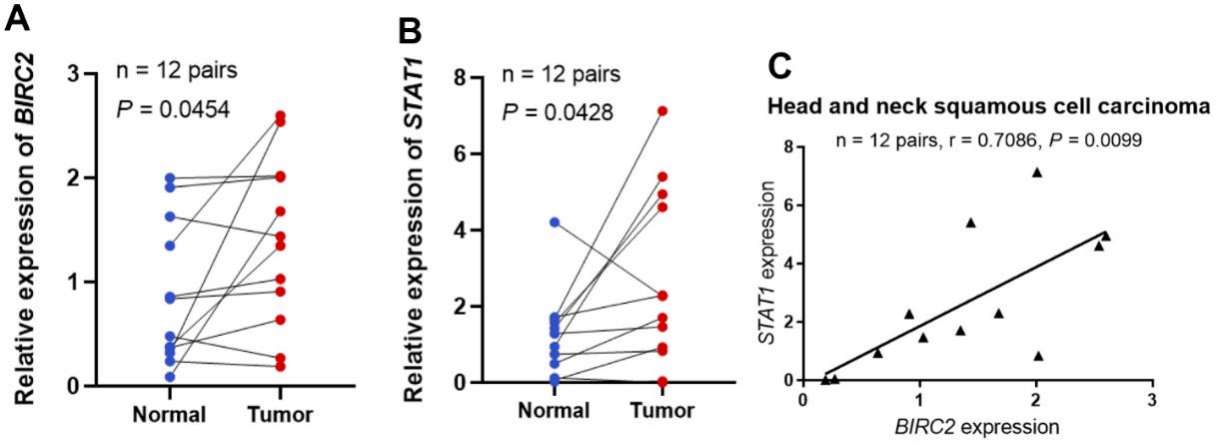
**STAT1 and BIRC2 expression levels in HNSCC.** (**A**) The BIRC2 mRNA expression levels in HNSCC. (**B**) The levels of STAT1 mRNA expression in HNSCC. (**C**) The correlations between the levels of STAT1 and BIRC2 mRNA expression.

The investigation revealed that BIRC2 might have a key role in promoting HNSCC progression. Additional experiments were carried out to validate the BIRC2 differential expression and its relationship with molecules linked to immune cells to elucidate its involvement in HNSCC. The BIRC2 expression in HNSCC samples was studied by qRT-PCR, and 12 HNSCC tissue specimens exhibited a much higher relative expression level of BIRC2 than corresponding nearby non-tumour tissue samples, according to the findings (*P* < 0.05; Figure 7A). Furthermore, we found a correlation between STAT1 and BIRC2, which is a marker gene for Th1 cells [19]. Relative to adjoining normal tissues, 12 HNSCC samples had a substantially higher relative expression level of STAT1, according to the qRT-PCR data (*P* < 0.05; Figure 7B). Lastly, we observed a strong positive correlation (R = 0.7086, *P* < 0.05; Figure 7C) after comparing the expression of BIRC2 and STAT1 in the 12 HNSCC samples.

**Figure 8:**
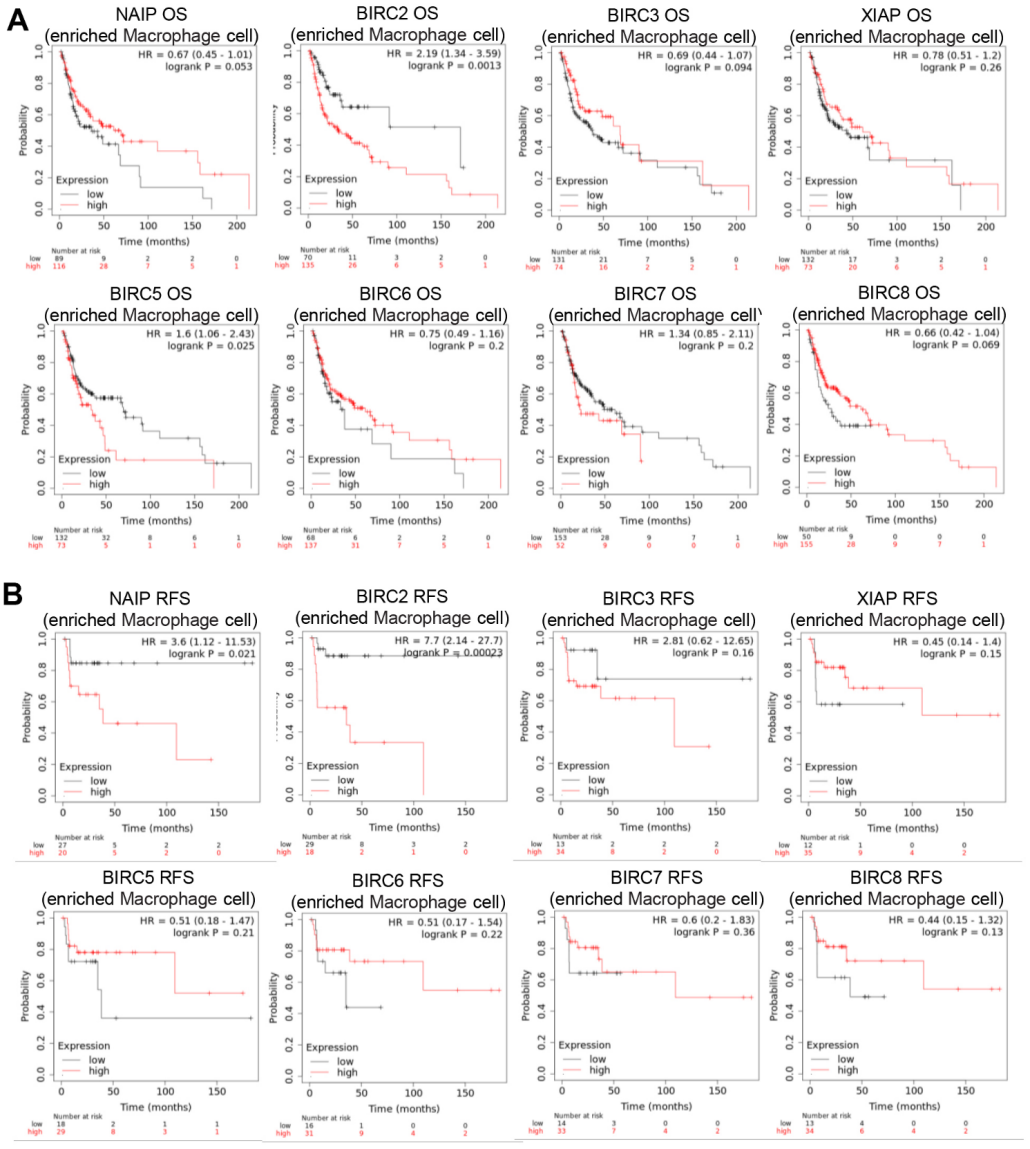
**Prognostic significance of IAP family member mRNA expression levels in HNSCC patients.** (**A**, **B**) Prognostic value of the IAP family in terms of macrophage cell enrichment in HNSCC.

The TME is the internal environment upon which tumour cells depend for survival. It comprises tumour cells, immune cells, chemokines, cytokines, and various other factors. TME is intimately linked with tumour growth, metastasis, immune evasion, and other behaviors. The main functions of Th1 cells are cellular immunity and inflammation. This includes activating other immune cells, like macrophages, B cells, and cytotoxic CD8+ T lymphocytes (CTL), promoting the targeted killing of infected cells, tumour cells, and abnormal cells, as well as inducing cell lysis and other effector functions. The IAP family may regulate macrophage polarisation in HNSCC patients, as certain members have a significant association with M2 macrophage markers. Consequently, the prognostic relevance of IAP family members in HNSCC patients with macrophage enrichment was explored. The findings indicate that in HNSCC patients, increased expression of BIRC2/5 negatively impacted the OS when there was macrophage enrichment (Figure 8A). In HNSCC patients with macrophage enrichment, higher expression of NAIP and BIRC2 was associated with poorer RFS (Figure 8B). These findings suggest that BIRC2 has a vital function in the infiltrating levels of the immune cells and the TME in HNSCC.

**Figure 9:**
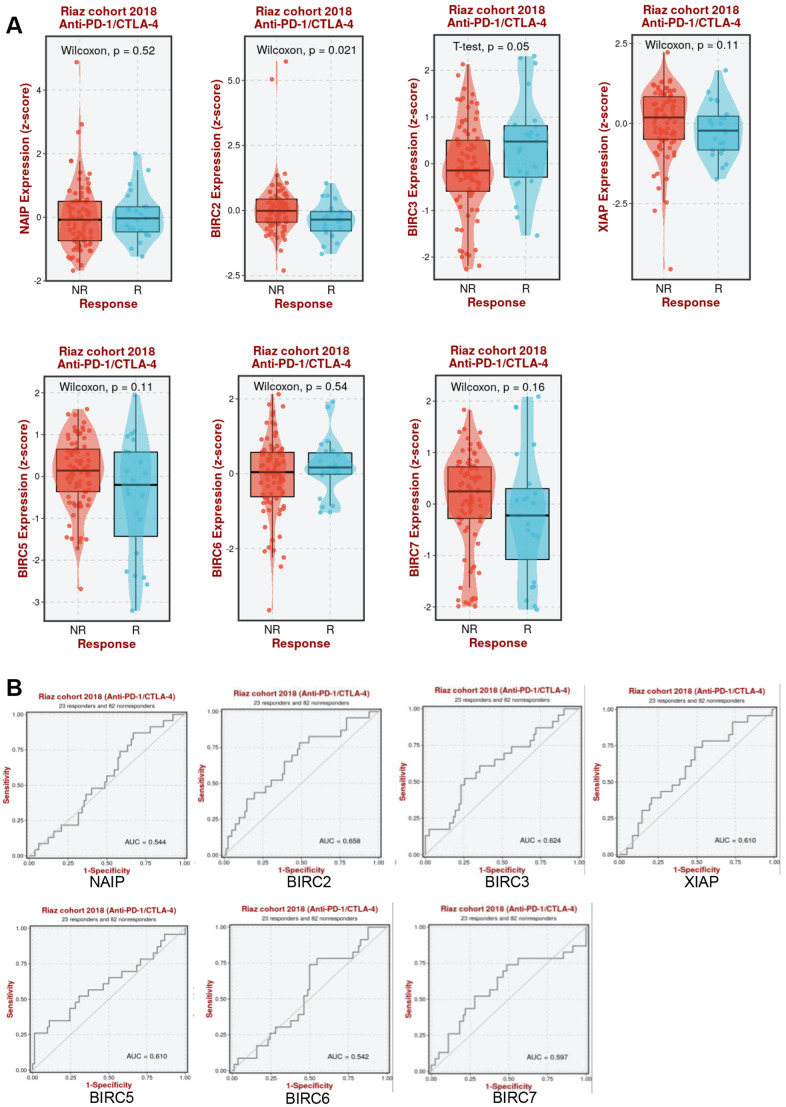
**Association between IAP family members and immunotherapy efficacy.** (**A**) Comparative analysis of the IAP family members levels in Riaz cohorts of anti-PD-1/CTLA-4 responders and non-responders. (**B**) Patients in the Riaz cohorts’ ROC curve for the IAP family.

It was further determined whether aberrant IAP family expression affects immunotherapy response in HNSCC. As shown in Figure 9A, BIRC2 expression increased in anti-PD-1/CTLA-4 non-responders in the Riaz cohort (*P* = 0.021). The Riaz cohort’s area under the receiver operating characteristic curve (AUC) was 0.658, implying that BIRC2 may effectively distinguish between individuals who respond to anti-PD-1/CTLA-4 and those who do not (Figure 9B). This study established BIRC2 as a promising immunotherapeutic target for HNSCC treatment.

## DISCUSSION

HNSCC is the most prevalent malignant tumour that occurs in the head and neck, and it originates from the mucosal epithelium of the larynx, pharynx, and oral cavity [20, 21]. There has been minimal indication of progress in the 5-year survival rates of HNSCC patients in recent decades, notwithstanding recent advancements in diagnosis and therapy [22]. Therefore, new and meaningful diagnostic and prognostic biomarkers and therapeutic targets are urgently required. The IAP protein encoded by the BIRC family gene is critical to the apoptosis resistance in various cancer cells [23–26]. However, there have been no systematic investigations into the role of the IAP family in HNSCC. We comprehensively elucidated the biological functions of IAP family members in HNSCC from five different aspects, namely, expression levels of mRNA and proteins, the disease’s pathological features and prognosis, pathway analysis, gene mutations, and immune infiltration. We discovered that the mRNA levels of all IAP family members were upregulated in HNSCC compared to those in non-tumour cells, but BIRC8 was not statistically significant. The expressions of NAIP, BIRC2/3/5/6, and XIAP protein levels were upregulated.

Inhibitors of apoptosis have been reported to have an increasingly significant function in the modulation of cell apoptosis, promotion of tumour development, and as a novel anti-cancer treatment strategy [27]. Inhibitors of apoptosis can regulate cell death not only by controlling caspases but also by modulating other signalling pathways that affect cell viability. One of the most significant contributions of IAPs to cell survival and tumourigenesis is their capacity to function as ubiquitin E3 ligases to regulate NF-κB signalling [28]. We probed the clinicopathological association and prognostic value of the aberrantly expressed members of the IAP family in HNSCC cases. A noteworthy link was identified between the expression of members of the IAP family and the staging, grading, and lymph node status of HNSCC. Upregulation of BIRC2/5/8 mRNA was accompanied by a worse OS in HNSCC, but upregulation of NAIP and BIRC2/7 was substantially linked to a worse RFS. The findings of this study indicate that this gene family, particularly BIRC2, could possess significant prognostic significance and serve as possible diagnostic markers for patients with HNSCC.

A growing body of research in recent years has illustrated a link between alterations in IAP expression and the onset and progression of tumours, as well as medication resistance. Researchers have found that BRCA mutations lead to XIAP overexpression, making ovarian cancer sensitive to the IAP family [12]. BIRC5 stimulates cellular apoptosis, diminishes the growth capacity of tumours, and renders cancerous cells more susceptible to chemotherapeutic agents (e.g., etoposide, Taxol, cisplatin), immunotherapy, and gamma radiation [29]. We further investigated IAP family mutations in HNSCC and found that BIRC2 and BIRC6 mutation rates were the highest (14 %). Epigenetic regulation may have a notable function in the biological processes of HNSCC that are induced by members of the IAP family. This suggests that the IAP family has the potential for use in new therapeutic interventions. Co-expressed genes belonging to the IAP family were analysed. Through enrichment analysis of the co-expressed genes, immunity-related signalling pathways, including cytokine-mediated signaling and the NF-B pathway, were shown to be substantially linked to IAP family members. The regulatory role of key molecules in immune-related pathways of HNSCC implies that the IAP family might potentially serve as a therapeutic target for this disease.

Immunotherapy has become a popular research topic in recent years [30, 31]. Cancer cells inhibit cell apoptosis by upregulating the expression of anti-apoptotic proteins, resulting in tumour growth, reduced prognosis, and resistance to drugs. Inhibitors of apoptosis have a pivotal role in regulating cell death via various mechanisms and act as significant regulators of cell death and survival signalling pathways. Dysregulation of IAPs is commonly linked with tumour growth and occurrence. There are two groups of IAPs, namely, Smac mimetics and non-peptide small molecules. Inhibitors of apoptosis serve not only as drug targets but can also be utilised as E3 ligases to design specific and non-genetic IAP-dependent protein eliminators, as well as to connect with other E3 ligands to synthesise heterobifunctional degraders [9, 32, 33]. Various studies demonstrate that IAP antagonists activate the NF-κB pathway, regulating innate and adaptive immunity, ultimately inducing anti-tumour activity [34]. Inhibitors of apoptosis antagonists, such as LCL161, have shown synergistic effects with anti-PD-1 immunotherapy [35]. These findings suggest that IAP antagonists hold potential as a newly developed immunomodulatory approach for cancer treatment in humans. Research indicates that the IAP family is implicated in the regulation of innate immune signalling and has a wide range of immunoregulatory features. It is therefore a suitable option for combination immunotherapies [36]. However, their role in HNSCC is not yet fully understood. Therefore, to identify promising targets for HNSCC immunotherapy, we examined the correlation between members of the IAP family and the infiltrating levels of immune cells. The analysis illustrated that the members of the IAP family had varying degrees of correlation with the most important immune cells. We found the levels of IAP family members (except BIRC5/8) were strongly correlated with macrophages. Therefore, found that macrophage-infiltrating BIRC2 can significantly improve RFS and OS in HNSCC patients. Based on these data, BIRC2 may be amenable to immunotherapeutic targeting in the context of HNSCC therapy. Consistent with our conclusions, the Riaz cohort demonstrated that anti-PD-1/CTLA-4 treatment significantly decreased BIRC2 levels in patients with HNSCC. It was shown that BIRC2 might better distinguish between immune responders and non-responders in HNSCC patients receiving anti-PD-1/CTLA-4 immunotherapy (AUC = 0.658). However, because the data analysed in this study were obtained from a network, there are limitations to the research. *In vitro* studies are necessary to verify the roles and potential mechanisms of the different IAP family genes in HNSCC.

## CONCLUSION

In conclusion, we elucidated the immunological and biological roles of the IAP family in HNSCC by systematically analysing IAP family members’ expression and prognostic potential in HNSCC using bioinformatic tools. Based on these results, BIRC2 could be a target and useful biomarker for personalised treatment of patients with HNSCC, which might contribute to the development of more effective ways of diagnosing and treating HNSCC, thereby improving patient outcomes. However, the mechanisms underlying their effect on the formation and progression of tumours and the development of drugs for their treatment remain unknown.

## MATERIALS AND METHODS

### Patients’ tissue samples

Twelve pairs of paraffin-embedded archival specimens of HNSCC and corresponding adjacent normal tissue samples were prospectively collected from Xiangya Hospital (Changsha, People’s Republic of China). The cohort included diverse HNSCC subtypes, specifically, four pairs of oral squamous cell carcinoma, one pair of nasopharyngeal squamous cell carcinoma, three pairs of tongue carcinoma, and four pairs of laryngeal carcinoma, along with their corresponding adjacent tissues.

### Exclusion and inclusion criteria

Inclusion and exclusion criteria were applied to identify eligible patients for this study. The inclusion criteria included: (1) histopathological confirmation of diagnosis; (2) no prior treatment with chemotherapy, radiation, or immunotherapy before resection; (3) the availability of complete data on clinical and pathological characteristics. The accompanying table offers a summary of the clinical parameters of the patients who were recruited. Exclusion criteria encompassed post-operative administration of alternative treatments, vital organ dysfunction, and the presence of secondary tumours in other organs.

### Quantitative real-time polymerase chain reaction (qRT-PCR)

RNA extraction, amplification, and qRT-PCR were executed using established protocols. The thermocycling protocol included an initial denaturation at 95° C for 30 seconds, followed by 40 cycles of annealing and extension phases at 60° C and 72° C, respectively, each lasting 30 seconds. The sequences of the primers utilised in qRT-PCR are highlighted in Table 2.

**Table 2:** Primer sequence for real-time PCR.

**Gene**	**Primer (Forward)**	**Primer (Reverse)**
BIRC2	AGCACGATCTTGTCAGATTGG	GGCGGGGAAAGTTGAATATGTA
STAT1	CGGCTGAATTTCGGCACCT	CAGTAACGATGAGAGGACCCT
U6	CTCGCTTCGGCAGCACA	AACGCTTCACGAATTTGCGT

### TIMER2.0

TIMER2.0 (https://cistrome.shinyapps.io/timer/) encompasses three primary components: immunisation, exploration, and estimation. This interactive tool enables the analysis of gene-immune infiltrating cell correlations, expression comparisons of genes between normal and malignant cells in a variety of cancers, and other functionalities, offering user-friendly interactive visualisations to facilitate data exploration [37]. The present work utilised the TIMER2.0 database to ascertain the expression patterns of IAP family members. Specifically, we evaluated the expression levels of these genes in 44 normal and 520 HNSCC samples. Additionally, using TIMER, we assessed the link between IAP family members’ mRNA expression and immune infiltrating cells in HNSCC, including CD4+ and CD8+ T lymphocytes, dendritic cells (DCs), neutrophils, macrophages, and B cells. The correlation analysis employed Spearman’s algorithm while adjusting for tumour purity. A *P* value < 0.05 was set as the significant threshold.

### GEPIA2

With a vast collection of 198,619 isoforms and covering 84 cancer subtypes, GEPIA2 (Gene Expression Profiling Interactive Analysis 2) provides an advanced platform for quantifying gene expression at the transcript level. It facilitates specific cancer subtype analysis and enables comparisons across different subtypes [38]. We utilised the GEPIA2 database to investigate the expression patterns of IAP family members within 44 normal samples and 520 samples of HNSCC.

### Human protein atlas (HPA)

The HPA portal (http://www.proteinatlas.org) serves as a valuable platform for a plethora of biomedical research projects, providing comprehensive protein expression data using immunohistochemistry (IHC) across different cancer types [39]. We implemented an in-depth evaluation of the protein expression profiles of IAP family members in both normal and HNSCC tissues utilising IHC techniques.

### UALCAN

UALCAN (https://ualcan.path.uab.edu) is an accessible and easily usable tool that offers readily available, pre-computed data for promoter DNA methylation status, protein/gene expression, and Kaplan-Meier (KM) survival analyses, organised by tumour subgroups [40]. Our stratified analysis focused on key patient-specific variables like cancer stage, tumour grade, and nodal status. Statistical significance was determined by the Student’s t-test, with a significance threshold of *P* < 0.05.

### Kaplan–Meier plotter database

The KM plotter database (https://kmplot.com) was utilised as a valuable resource for conducting a meta-analysis, integrating clinical prognostic data with gene expression data to detect and confirm survival-related molecular markers [41]. We utilised the KM plotting approach to explore the link between RFS and OS with the IAP family members in HNSCC patients. The KM analysis was evaluated using the log-rank test, and a *P* < 0.05 was set as the significance threshold.

### CBioPortal

The cBio Cancer Genomics Portal (http://cbioportal.org) allows users to interactively explore multidimensional cancer genomic information on a publicly available platform [41, 42]. We leveraged the capabilities of cBioPortal to obtain a dataset comprising 523 patients diagnosed with HNSCC. Subsequently, the IAPs were analysed using this dataset for co-expression and gene alterations.

### STRING

The STRING database (https://cn.string-db.org/) serves as a comprehensive resource offering information regarding predicted and known protein-protein associations across a wide range of organisms. Physical interactions and functional linkages are both included in this comprehensive database, along with confidence levels indicating their reliability [43]. In our study, we harnessed the capabilities of the STRING database to evaluate the correlations among IAP genes.

### Cytoscape

Cytoscape (http://cytoscape.org) is a publicly accessible program that aims to build biomolecular interaction networks that can be easily integrated with other molecular states and high-throughput expression data [44]. Within the scope of this study, Cytoscape was utilised to enable the functional integration of 156 frequently altered genes belonging to the IAP family, which were identified through screening the cBioPortal database. The degree values, reflecting the interactions between these proteins, were visually represented by the size of the nodes in the network, with larger circles denoting proteins exhibiting higher degrees of connectivity.

### Metascape

Membership search, gene annotation, interactome analysis, and functional enrichment, are all synergistically integrated into Metascape (https://metascape.org), an all-inclusive and integrated portal. It harnesses the collective knowledge from over 40 independent databases to provide a robust analytical framework [45]. In this study, we utilised GO and KEGG pathway enrichment analyses to ascertain the functional implications of the IAP family.

### Biomarker Exploration of Solid Tumours (BEST)

The study’s findings were validated by utilising the BEST portal, an established and reputable platform for comprehensive biomarker analysis in the field of oncology research (https://rookieutopia.com/app_direct/BEST/). Patients with HNSCC were studied using BEST to determine whether there was a link between the IAPs and responsiveness to immunotherapy as well as the prognostic outcomes.

### RNA isolation from the Formalin-Fixed and Paraffin-Embedded (FFPE) samples

FFPE colon cancer or normal tissue samples were subjected to deparaffinisation using xylene. Total RNA isolation from these samples was performed using the AmoyDx® FFPE RNA Extraction Kit for total RNA (Cat. # 8.02.0019, AmoyDx, Xiamen, P. R. China).

### Statistical analysis

Survival analysis was conducted via the log-rank test, while Spearman’s correlation was employed to evaluate the connection between the IAP family and indicators of immune cell type and immunological infiltration. The two independent samples were contrasted utilising Student’s t-test. *P* < 0.05 was set as the significance criterion.

### Data availability statement

The original contributions presented in the study are included in the article/Supplementary Material, further inquiries can be directed to the corresponding authors.

## Supplementary Material

Supplementary Table 1
